# Do upper leg compression garments aid performance and reduce exercise-induced muscle damage in recreational marathon runners?

**DOI:** 10.17159/2078-516X/2022/v34i1a14169

**Published:** 2022-01-01

**Authors:** KM Kabongo, A Emeran, AN Bosch

**Affiliations:** 1 Division of Physiotherapy, Department of Human Biology, Department of Health and Rehabilitation Sciences, Faculty of Health Sciences, University of Cape Town, Cape Town, South Africa; 2 UCT Research Centre for Health through Physical Activity Lifestyle and Sport (HPALS), Department of Human Biology, Faculty of Health Sciences, University of Cape Town, Cape Town, South Africa; 3 International Federation of Sports Medicine (FIMS) Collaborative Centre of Sports Medicine, University of Cape Town, Cape Town, South Africa

**Keywords:** endurance running, recovery, muscle soreness, compression shorts

## Abstract

**Background:**

Despite the lack of scientific knowledge on the physiological and biomechanical effects of wearing compression garments (CGs), there has been an increase in the use of compression garments (CG) amongst endurance runners.

**Objectives:**

To compare marathon race performance, post-race pain, and mid-thigh circumference in marathon runners using upper leg CGs, with runners who did not use CGs in the same marathon race.

**Methods:**

The study was conducted on healthy, long-distance runners (n=18) participating in the Winelands Marathon race, Cape Town, South Africa. The CG group (n=10) participated in the race wearing upper leg CGs, while the control group (n=8) did not. Participants were tested on three occasions for various subjective markers of exercise-induced muscle damage (Visual analogue scale (VAS) pain rating score, and Likert scale for muscle pain), mid-thigh circumference for muscle swelling, and running performance (race pace).

**Results:**

VAS pain ratings for hamstring (p=0.04), knee flexion (p=0.02) and hip extension (p=0.04) were significantly lower than the ratings of the control group immediately post-race and two days post-race. No statistically significant differences were detected in race performance, mid-thigh circumferences or Likert scale for determination of muscle soreness.

**Discussion:**

Wearing of upper leg CGs while running a marathon race improved VAS pain ratings immediately post-race through to two days post-race. However, due to no placebo control, this beneficial effect may be psychological as opposed to a physiological effect of the CGs on muscle pain.

**Conclusion:**

The use of upper leg CGs reduced subjective muscle pain in runners in the first 48 hours post-race.

Marathon running has become increasingly popular and more competitive in recent years, due to its easy accessibility, its associated health benefits, and the popularity of iconic races.^[1]^ An example of one such iconic and increasingly popular race is the Boston marathon, with the number of marathon finishers increasing from 1 848 in 1972 to 26 657 in 2019.^[2]^

Despite the health benefits of long-distance running, many runners experience exercise-induced muscle soreness post training. Most distance running racecourse profiles, such as the marathon, contain a variety of uphill, downhill, and flat sections. The downhill sections have the greatest effect on the lower extremity, as it induces a high proportion of eccentric muscle action on the quadricep muscles of the upper leg.^[3]^ Eccentric exercise, even in trained runners can result in exercise-induced muscle damage (EIMD) resulting from mechanical damage to the sarcomeres. This mechanical damage leads to an inflammatory response, which is proposed to exacerbate the degree of damage.^[4]^ The signs and symptoms of EIMD include temporary reductions in muscle strength, decreased rate of force development (power), reduced range of motion, swelling, increased feelings of soreness and the appearance of intracellular proteins in the blood.^[4]^

In recent years strategies to reduce symptoms of EIMD and improve recovery processes have been investigated and implemented to improve performance of marathon athletes.^[5]^ One such method is the use of compression garments (CGs) to improve performance and aid recovery in marathon runners.^[6]^

There is limited knowledge on the mechanisms underpinning the efficacy of CG usage in marathon runners, although there are several hypotheses on possible mechanisms. These include a reduction in muscular microtrauma and tissue vibration during exercise, and reduced muscle fibre recruitment when utilising CGs.^[7]^ It has also been proposed that CGs are effective in reducing the swelling and inflammatory processes associated with muscle damage. It is theorised that the CGs work by creating an external pressure gradient that reduces the space available for swelling to occur, thereby reducing the secondary inflammatory responses.^[8]^ Another theory is that the CGs improve venous return, reduce venous pooling, and promote the removal of metabolites, due to the muscle pump function.^[9]^

The use of CGs by runners remains a controversial issue as there is a wide variety of findings in studies, often contrasting one another.^[7]^ Furthermore, as far as we are aware, most studies have investigated the use of compression socks and tights on performance and recovery, with few studies investigating the use of upper leg CGs specifically.^[7]^

The aim of this study was therefore to assess the effect of upper leg CGs on performance and EIMD in marathon runners using upper leg CGs in a marathon race, compared to runners who did not use CGs in the same race. Subjective pain and mid-thigh circumference (an indirect measure for muscle swelling and inflammation)^10^ were used as indirect measures of muscle damage.

## Methods

### Study design and participants

Eighteen recreational marathon runners volunteered to participate in this randomised controlled intervention study. Inclusion criteria required the participants to be free from lower limb musculoskeletal injuries for the previous three months prior to participation, pass a Physical Activity Readiness Questionnaire (PAR-Q) and medical clearance. They were required to be 20–45 years old, be participating in the Winelands Marathon (42.2km), have a minimum average training distance of 50km per week and to have completed at least one marathon in the preceding 18 months. Furthermore, any participants who had routinely used CGs and/or who were unwilling to train and/or compete with or without CGs based on the potential group allocation, were excluded.

The participants were initially matched based on their sex, age, and personal best marathon time. They were then randomly allocated to either a CG group (n=10) wearing CGs or a control group (n=8). Both groups were instructed to train as usual and record their training. The CG group participated in the race while wearing upper leg CGs. The control group were instructed to neither train nor participate in the race with CGs.

### Ethical considerations

The study was conducted on the principles of the Declaration of Helsinki (2013). Ethical approval was approved by the University of Cape Town (UCT) Human Research Ethics Committee (HREC) (HREC: 208/2019). Written informed consent was obtained from all participants prior to their participation.

### Data collection

Testing and data collection occurred on three separate occasions. The initial data collection (Visit 1) was performed three days before the marathon. Mid-thigh circumference was recorded. Body mass was measured with a calibrated digital scale and stature was measured with a stadiometer to calculate the body mass index (BMI). Body fat percentage was calculated after measuring the skinfold thickness of four sites: Biceps, Triceps, Subscapularis and Suprailiac Crest, with a Harpenden skinfold caliper.^[11]^ These data are reported in Table 1.

The second data collection (Visit 2) was performed directly after the completion of the Winelands Marathon. The mid-thigh circumference measurements were repeated at this visit and the participants completed a Likert Scale for determination of muscle soreness, as well as a Visual Analogue Scale (VAS) pain ratings questionnaire.

The final data collection (Visit 3) was performed two days following the marathon, with the mid-thigh circumference measurements, the Likert Scale, and VAS pain rating questionnaires being repeated. The race performance results of the participants were retrieved electronically, and participants' finish times and average race pace were recorded. In addition, a questionnaire was completed to assess CG utilisation, nutrition and fluid strategies during the race, recovery modalities used, training details for a period of six weeks prior to the marathon, and menstrual cycle for females (to account for any possible confounding variables during the race).

### Mid-thigh circumference

The mid-thigh circumference was measured to estimate post-race exercise-induced muscle swelling.^[11]^ One commonly used method for circumference measurements is using a tape measure, as it is inexpensive, efficient, rapid, and reliable.^[12]^ The participants’ measurements were obtained without CGs and taken midway between the trochanterion and lateral border of the tibia, at the mid-trochanterion-tibiale laterale site (according to the International Standards for anthropometric assessment).^[13]^ Each measurement was performed three times and the average value reported.

### Visual analogue scale for pain ratings

A VAS questionnaire was administered to determine subjective pain ratings on a scale of 0 – 10 and was used in conjunction with non-weight bearing active movements of the hip and knee. Zero represented no pain and 10 represented severe pain. The construct validity of the numerical version of the VAS has been recorded as high as 0.91.^[14]^ The participants were required to mark on the line the point that they felt represented their perception of pain. The VAS score was determined by measuring in millimetres from the left-hand end of the line to the point that the participants marked.

### Likert scale for determination of muscle soreness

The Likert scale for the determination of muscle soreness was administered for subjective pain rating based on a six-point system. Zero represented a complete absence of muscle soreness and 6 represented severe pain that limited the ability to move. The participants ticked what best described their muscle soreness.

### Race time

FinishTime timing chips are internationally recognised, highly reliable and validated and was the official timing tool used for the Winelands Marathon (finishtime.co.za).^[15]^ The time to complete the race was obtained from the online results and used to calculate the overall average running pace (min:sec/km) during the race.

### Compression garment composition

Commercially available, graded upper leg CGs produced internationally (China) were used in the study. The composition of the garments was 55% nylon, 40% polyester and 5% elastane fabric. The manufacturers of the CGs were not involved in financing the study. The pressure exerted by the CGs on the leg was not measured.

### Statistical analysis

All statistical analyses were performed using SAS software version 9.4 (2020) and Microsoft Excel software (2019). The confidence intervals were set at 95% and statistical significance was determined as p<0.05.

Due to data not being normally distributed, a nonparametric approach for analysis was used. For race performance, mid-thigh circumference, VAS pain rating, Likert scale for the determination of muscle soreness, descriptive data (age, height, body mass, BMI, body fat percentage and previous best marathon time) and for the questionnaires, the Mann-Whitney U test was used to compare the groups. To determine differences in previous training history and recovery modalities, a Fisher's exact test was performed. To determine whether there was a difference in the frequency of use of CGs prior to the study, the CG frequency scale was compared between the groups using a Cochran-Armitage test for trend. In addition, a Hodges-Lehmann 95% confidence interval for the median difference was calculated.

## Results

Runners were recruited by an email sent to all Winelands Marathon 42.2km entrants aged between 20 and 45 years. Forty responses were received with all 40 being eligible to participate. After the screening process, one participant was excluded. A further 19 participants withdrew from the study before the first data collection, with the majority withdrawing due to being too busy to attend the sessions, work commitments, transport issues, change of plans and deciding to race the half marathon (21.2km) instead. Thereafter, there were 10 participants in each group. A further two participants withdrew during the data collection process at Visit 2, and one participant in the control group injured his hamstring during the race and was unable to complete the race. A second participant in the CG withdrew due to logistical issues, as he did not report for follow-up visits. Only participants who completed all three visits of the data collection process were included in the final reporting of the study.

No statistically significant differences were observed in the baseline descriptive data of the two groups (Table 1).

### Race performance

The race pace was utilised to measure the performance of the participants (from start to finish of the 42.2km marathon race). The CG group had an average race pace of 6:11 min:sec/km compared to 6:44 min:sec/km of the control group, which was not statistically significantly different (p=0.27). Both groups had a slower running pace (min/km) compared to their personal best times (Table 1). To adjust for the relative effort of each runner relative to their best marathon time, the difference in running pace (min/km) between the Winelands marathon and personal best was calculated. However, no statistically significant differences were found between the groups (p=0.69).

### Mid-thigh circumference

**Fig. 1:**
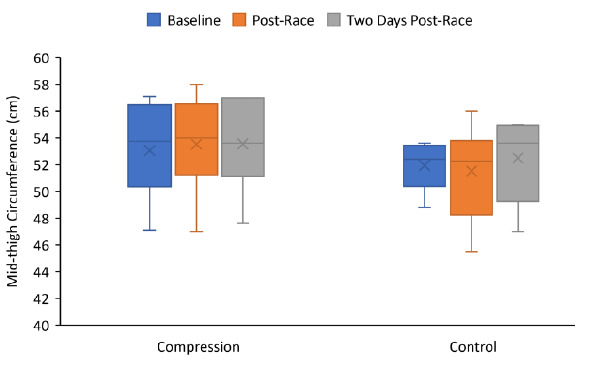
Box and whiskers plot of Mid-thigh circumference by visit for the CG (n=10) and control (n=8) groups. The box indicates the median and the interquartile range (quartile 1 to 3). The “x” represents the mean. The “whiskers” indicate the range of the data.

The CG group’s median mid-thigh circumference increased slightly (0.20cm) from baseline testing (53.8cm) to immediately post-race (54.0cm) compared to the control group which recorded a 0.15cm decrease from baseline (52.4cm) to immediately post-race (52.3cm). Two days post-race the CG group median mid-thigh circumference decreased from 54.0cm to 53.6cm and the control group increased from 52.4cm to 53.6cm. However, none of these changes were statistically significant (p=0.37). The absolute mid-thigh circumference measurement changes over time are presented in Figure 1.

### VAS pain ratings

Immediately at the completion of the race and two days post-race, the VAS pain ratings were recorded for the hamstring and quadricep muscles at rest, during hip flexion and extension, and knee flexion and extension. Both groups decreased in median VAS pain rating scores from immediately post-race to two days post-race. There were statistically significant differences in VAS pain scale rating scores during knee flexion (p=0.02), resting hamstring (p=0.04), and during hip extension (p=0.04) for both legs immediately post-race and two days post-race, with the CG group having lower VAS pain ratings compared to the control group (Table 2).

### Likert scale for the determination of muscle soreness

**Fig. 2:**
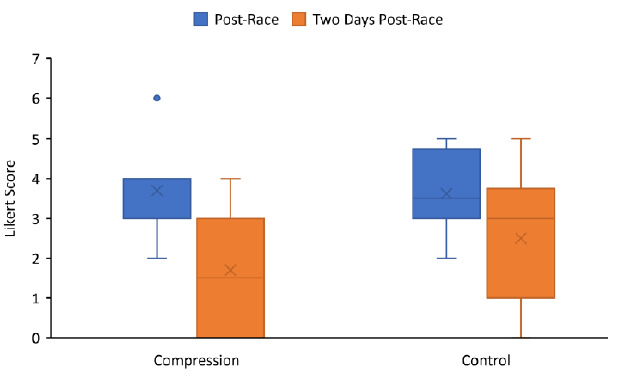
Box and whiskers plot of Likert scale score by visit for the CG (n=10) and control (n=8) groups. The box indicates the median and the interquartile range (quartile 1 to 3). The “x” represents the mean. The “whiskers” indicate the range of the data. The dots represent outlier values. The Likert scale indicates subjective muscle soreness based on a six-point system where 0 represented a complete absence of muscle soreness and 6 represented severe pain that limited the ability to move.

A Likert scale for the determination of muscle soreness scores was recorded immediately post-race and two days post-race. Although the muscle soreness score decreased in both groups from post-race to two days post-race, with a greater decrease in the CG group compared to the control group, these changes were not statistically significant (p=0.46). The absolute change in median Likert scale for determination of muscle soreness is presented in Figure 2.

### Questionnaire

There were no statistically significant differences (p>0.05) found in self-reported data for training history prior to the Winelands Marathon, CG utilisation, muscle recovery strategies utilised, nutritional and fluid intake during the race and menstrual cycle for females.

## Discussion

This study compared the performance, pain and mid-thigh circumference changes in marathon runners using upper leg CGs against runners who did not use CGs in the same marathon. The main findings were that CGs resulted in VAS pain ratings which were statistically significantly better post-race compared to the control group. However, there were no statistically significant improvements in race time, mid-thigh circumference measurements or Likert scale score for muscle soreness post-race.

### Race performance

No difference in race performance was observed between groups (p=0.27), indicating that the CGs did not improve running performance. This finding concurs with other studies that observed no improvement in race running times and endurance performance when wearing lower limb CGs.^[7,16,17]^ In contrast, some studies testing lower limb CGs in runners during incremental and step tests, found small positive effects on time to exhaustion.^[7]^

### Mid-thigh circumference

It has been proposed that CGs could reduce exercise-induced swelling and potential inflammation associated with EIMD^[8,9]^, which is theorised to cause an increase in overall mid-thigh circumference.^[8]^

Although the control group had an overall increase in mid-thigh circumference at two days post-race, while the CG group had a decrease at two days post-race, none of these changes were statistically significant. (p=0.37).

Similarly, a study by Geldenhuys et al.^[16]^ found no statistically significant changes in the calf circumference of runners wearing CGs post-ultramarathon race. However, a smaller increase in ankle circumference post-race to two days post-race in a CG group, compared to a control group (p=0.01) was reported.^[16]^ This is similar to our study, where the mid-thigh circumference of the control group increased more than the CG group at two days post-race, albeit not statistically significant.

Thus, the results of this study do not support the theory of CGs reducing swelling and inflammation through changes in mid-thigh circumference. The use of biochemical markers of inflammation may be a more accurate, albeit a more invasive method of measuring inflammatory changes post-exercise. However, a study by Pruscino et al. found no differences in inflammatory markers when a lower CG was worn after intermittent exercise, compared to a control group. ^[17]^

### VAS pain rating scale

The main findings of the current study were found within the VAS pain rating outcome measure, with several statistically significant findings in the CG group, including a lower resting hamstring VAS pain rating score at rest, on hip extension and knee flexion immediately post-race and two days post-race, compared to the control group. Thus, the CGs were associated with a reduction in subjective pain for a period of 48 hours post-completion of a marathon.

These findings are similar to the results of previous studies,^[4,7]^ in which muscle soreness was reduced in runners when utilising CGs, but contradict the findings of Geldenhuys et al., who reported a higher VAS pain rating score for participants wearing below-knee compression garments post-ultra-marathon, compared to a control group.^[16]^

Based on the findings of the current study, it is suggested that the use of upper leg CGs during running may be beneficial as it may assist with recovery in the first 48 hours post-race. However, the practical importance of this may be limited, unless it extends to long distance training runs, as runners who have participated in a marathon race will move into a recovery phase of training, and thus it is of limited importance whether the wearing of CGs during a race reduces post-race EIMD and improves recovery. Speeding up recovery from long training runs, however, could be very useful in an athlete attempting to maximise training load.

### Likert scale

In addition to the VAS pain rating, an additional subjective pain scale (the Likert scale) was used to assess pain scores. The Likert scale assessed functional activities compared to the VAS pain rating which assessed static and dynamic movements of the hamstring and quadricep muscle. To our knowledge this is the first study to utilise this subjective assessment of muscle soreness in the study of CGs. It would be anticipated that the CG group would report lower scores, as suggested by the VAS pain ratings. However, there were no statistically significant findings detected between the groups in muscle soreness based on the Likert scale.

### Study limitations

There are several limitations of the current study, including a small sample size, using subjective measures of muscle damage (pain scales) and indirect measures of muscle swelling (mid-thigh circumference), that may not be as accurate as objective measures. Although the pressure exerted by the CGs on individual participants was not measured and may be seen as a limitation, several studies have found no apparent association between pressure applied and CG effects, as beneficial effects have been observed in both high and low pressure CGs.^[19]^

A post-race performance test was not performed, which could have been a direct measure of recovery. Furthermore, the study did not control for a possible placebo effect. Thus, results should be taken with caution, with the possibility that the greater reduction in VAS pain ratings in the CG compared to the control group could be a psychological benefit as opposed to physiological.^[17]^

## Conclusion

In conclusion, the wearing of upper leg CGs while running a marathon race did not improve race performance, change post-race mid-thigh circumference, or improve Likert scores for muscle pain. However, reduced VAS pain ratings were reported post-race for the hamstring at rest, knee flexion and hip extension movements, compared to a control group. Based on the findings, there is indication from some of the measures used that the use of upper leg CGs reduces subjective muscle pain in runners in the first 48 hours post-race, which may aid in recovery. The lack of a performance benefit, and the small improvement in self-reported pain post-race suggests that it is probably of limited value to use upper leg CGs during a race.

## Figures and Tables

**Table 1:** Demographic, body composition and training descriptive data of the CG and control groups

	CG (n=10)	Control (n=8)	p-value
**Males (n)**	6	6	-
**Females (n)**	4	2	-
**Age (years)**	38 (28–42)	38 (36–39)	1.00
**Body mass Visit 1 (kg)**	74 (64–89)	77 (69–80)	0.97
**Body mass Visit 2 (kg)**	72 (63–86)	74.70 (66–78)	0.97
**Body mass Visit 3 (kg)**	74 (62–89)	77 (68–80)	0.97
**Height (cm)**	172 (167.75–177.50)	174 (167.88–177)	0.31
**Body fat (%)**	24 (22–30)	23 (21–27)	0.66
**BMI (kg/m** ^**2**^ **)**	25.70 (23.70–26.40)	24.90 (22.90–26.70)	0.86
**Pace in PB marathon in past 18 months (min:sec/km)**	05:44 (04:51–06:18)	05:49 (05:39–06:48)	0.63

Data are expressed as median (interquartile range) unless stated otherwise.CG, compression garment group; BMI, body mass index; PB, personal best

**Table 2:** VAS pain ratings immediately post-race and two days post-race for the CG and control groups

	Immediately post-race						Two days post-race					
	CG		Control		p-value		CG		Control		p-value	
	Right	Left	Right	Left	Right	Left	Right	Left	Right	Left	Right	Left
**Quadriceps at rest**	2.50 (2.00–4.00)	2.50 (2.00–4.00)	2.00 (1.00–3.00)	2.00 (1.00–3.00)	0.69	0.69	0.00 (0.00–1.00)	0.00 (0.00–1.00)	1.00 (0.00–2.00)	1.00 (0.00–2.00)	0.69	0.69
**Hamstring at rest**	2.50 (0.00–4.00)	2.50 (0.00–4.00)	4.00 (2.50–4.50)	4.00 (2.50–4.50)	0.04*	0.04*	0.00 (0.00–1.00)	0.00 (0.00–1.00)	1.00 (0.00–3.00)	1.00 (0.00–3.00)	0.04*	0.04*
**During knee flexion**	2.50 (1.00–5.00)	2.50 (1.00–5.00)	5.00 (3.50–7.00)	5.00 (3.50–7.00)	0.02*	0.02*	1.00 (0.00–2.00)	1.00 (0.00–2.00)	2.00 (2.00–3.50)	2.00 (2.00–3.50)	0.02*	0.02*
**During knee extension**	2.50 (1.00–4.00)	2.50 (1.00–4.00)	3.50 (2.00–4.50)	3.50 (2.00–4.50)	0.21	0.21	0.50 (0.00–2.00)	0.50 (0.00–2.00)	1.50 (1.00–2.00)	1.50 (1.00–2.00)	0.21	0.21
**During hip flexion**	3.50 (2.00–4.00)	3.50 (2.00–4.00)	3.00 (2.00–4.50)	3.00 (2.00–4.50)	0.33	0.33	1.00 (0.00–2.00)	1.00 (0.00–2.00)	2.5 (1.00–4.00)	2.5 (1.00–4.00)	0.33	0.33
**During hip extension**	2.50 (2.00–4.00)	2.50 (2.00–4.00)	4.00 (2.00–5.50)	4.00 (2.00–5.50)	0.04*	0.04*	1.00 (0.00–2.00)	1.00 (0.00–2.00)	2.5 (2.00–3.50)	2.5 (2.00–3.50)	0.04*	0.04*

Data are expressed as median (interquartile range) unless stated otherwise. VAS pain rating is on a scale of 0 to 10 where 0 is no pain and 10 is severe pain. Right and left refers to the side or limb where the VAS pain rating was recorded.

* indicates p<0.05;

CG, compression garment group.
